# High expression of cholesterol biosynthesis genes is associated with resistance to statin treatment and inferior survival in breast cancer

**DOI:** 10.18632/oncotarget.10746

**Published:** 2016-07-21

**Authors:** Siker Kimbung, Barbara Lettiero, Maria Feldt, Ana Bosch, Signe Borgquist

**Affiliations:** ^1^ Division of Oncology and Pathology, Department of Clinical Sciences, Lund University, Lund, Sweden; ^2^ Department of Oncology, Skåne University Hospital, Lund, Sweden

**Keywords:** statin, breast cancer, cholesterol biosynthesis, predictive biomarker, prognosis

## Abstract

There is sufficient evidence that statins have a protective role against breast cancer proliferation and recurrence, but treatment predictive biomarkers are lacking. Breast cancer cell lines displaying diverse sensitivity to atorvastatin were subjected to global transcriptional profiling and genes significantly altered by statin treatment were identified. Atorvastatin treatment strongly inhibited proliferation in estrogen receptor (ER) negative cell lines and a commensurate response was also evident on the genome-wide transcriptional scale, with ER negative cells displaying a robust deregulation of genes involved in the regulation of cell cycle progression and apoptosis. Interestingly, atorvastatin upregulated genes involved in the cholesterol biosynthesis pathway in all cell lines, irrespective of sensitivity to statin treatment. However, the level of pathway induction; measured as the fold change in transcript levels, was inversely correlated to the effect of statin treatment on cell growth. High expression of cholesterol biosynthesis genes before treatment was associated with resistance to statin therapy in cell lines and clinical biopsies. Furthermore, high expression of cholesterol biosynthesis genes was independently prognostic for a shorter recurrence-free and overall survival, especially among ER positive tumors. Dysregulation of cholesterol biosynthesis is therefore predictive for both sensitivity to anti-cancer statin therapy and prognosis following primary breast cancer diagnosis.

## INTRODUCTION

There is a continuous search for complementary treatments, which can act in concert with already approved anti-cancer therapeutics to prevent or delay the development of therapy resistance and prolong the survival of patients diagnosed with cancer. In breast cancer, statins have received recognition as potential anti-cancer agents because several epidemiological studies have confirmed an association between statin use and a decreased risk of disease recurrence following adjuvant treatment [1–4]. Pre-clinical studies also indicate that statins can decrease breast cancer cell growth and induce apoptosis in various experimental models [5–8]. Furthermore, we and others have reported results from pre-surgical “window-of-opportunity” clinical trials in breast cancer confirming that statins can decrease tumor proliferation and induce apoptotic cell death [9, 10]. Not surprising, a heterogeneous response to statin treatment was seen across cell lines and primary tumors [5, 6, 8–10]. Today, breast cancer molecular and clinical heterogeneity is unequivocally acknowledged and treatment is tailored to specific subtypes of the disease. An important goal of oncological therapy is to maximize treatment efficacy while minimizing toxicity and over-treatment. Considering the potential benefits of adding statins (which are well tolerated, safe and inexpensive drugs) to established breast cancer therapeutic regimens, there is a need for clinical trials to prospectively evaluate the role of statins in breast cancer therapy. Hence, treatment predictive markers to select patients most likely to derive benefit from statin therapy will be of great value to effectively design such studies.

Previous investigations to distinguish the molecular features between statin sensitive and insensitive multiple myeloma cell lines revealed that dysregulation of the mevalonate pathway was a key determinant of atorvastatin sensitivity in this model [11]. A similar investigation in breast cancer cell lines showed that fluvastatin sensitivity was associated with the absence of ER expression and the basal-like molecular subtype [6]. Herein, we validate the association between atorvastatin sensitivity and the dysregulation of cholesterol biosynthesis in breast cancer. Further, we present a novel multigene signature, comprised of genes involved in cholesterol biosynthesis, with the potential to differentiate breast cancer cell lines and primary tumors according to their sensitivity to statin treatment. Furthermore, we show that this multigene signature is significantly differentially expressed in cell lines and primary tumors, and is an independent prognostic factor following primary breast cancer diagnosis, especially in patients with ER positive tumors.

## RESULTS

### Atorvastatin treatment differentially induces the cholesterol biosynthesis process in breast cancer cells

Initially, we investigated the effect of atorvastatin treatment on the proliferation rate of the four selected breast cancer cell lines, representing different breast cancer molecular subtypes. MDA-MB-231 (ER-/PR-/HER2-) cells were extremely sensitive to statin treatment with less than 30% of cells surviving after 72 hours exposure to 2 μM of atorvastatin (Supplementary Figure S1A). SKBR3 (ER-/PR-/HER2+) cells also displayed moderate sensitivity to the treatment, requiring a higher dose of 5μM to keep the proliferation rate below 30% at 72 hours (Supplementary Figure S1A). MCF7 (ER+/PR+/HER2-) and BT474 (ER+/PR+/HER2+) were less sensitive to atorvastatin treatment requiring doses >20 μM to keep the proliferation rates under 30% at 72 hrs (Supplementary Figure S1A). This data suggests that cells lacking the expression of the ER may be more sensitive to statin treatment.

**Figure 1:**
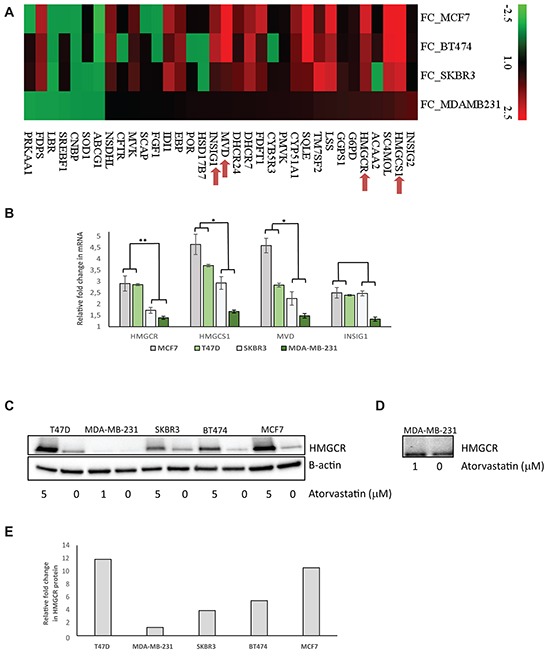
Atorvastatin treatment differentially induces the expression of genes in the cholesterol biosynthesis process in breast cancer cell lines **A.** Fold change of mRNA expression for significantly altered cholesterol biosynthesis genes after 48 hours atorvastatin treatment. Red in the heat map represents upregulation and green represents downregulation. **B.** RT-qPCR validation of the statin-induced feedback upregulation of four selected cholesterol biosynthesis genes. Fold change of mRNA expression after 48 hours of atorvastatin treatment are shown. The average fold change in each gene was compared between the less sensitive (MCF7 and T47D) and the sensitive (SKBR3 and MDA-MB-231) cell lines. * represents P<0.05 and ** represents P<0.01 respectively. **C.** A corresponding upregulation of HMGCR protein following atorvastatin treatment is also demonstrated in the respective cell lines. **D.** Following a prolonged exposure of the western blot in (C) for image acquisition, the expression of HMGCR in MDA-MB-231 cells become clearly visible, showing only a modest increase in protein expression after atorvastatin treatment. **E.** Relative quantification of the fold difference in HMGCR protein abundance between the controls vs atorvastatin treated cell lysate in each respective cell line.

However, other ER independent transcriptional features may exist, which could distinguish statin sensitive cells from the insensitive cells. As reported in our previous study [12], atorvastatin significantly perturbed the transcriptome of the MDA-MB-231 and SKBR3 cells but had very minor effects on the MCF7 and BT474, greatly mirroring the effects on cell proliferation. Specifically, in MDA-MB-231 and SKBR3 cells, several gene transcripts encoding proteins regulating key cellular processes including DNA replication and progression through the cell cycle were significantly down-regulated while a significant upregulation of genes involved in cell cycle arrest and induction of programmed cell death was seen (Supplementary Figure S1B and [12]). Importantly, a subset of genes involved in the cholesterol biosynthesis pathway was found to be statistically significantly upregulated by atorvastatin treatment in all cell lines, irrespective of the level of sensitivity to the treatment (Supplementary Figure S1B and [12]). In-depth inspection revealed that although the biological process “cholesterol biosynthesis” was statistically significantly upregulated in all cells lines, differences in the magnitude of the fold change in the transcript levels of the deregulated genes existed between the cell lines (Figure 1A). Higher fold changes were observed in MCF7 and BT474 relative to SKBR3 and MDA-MB-231, the magnitude of which was inversely correlated with the effects of the treatment on cell proliferation. Amongst the upregulated genes, was the molecular target of statins, *HMGCR*. Similarly, using RT-qPCR, the statin-induced feedback up-regulation of four selected cholesterol biosynthesis genes (*HMGCR, HMGCS1, MVD* and *INSIG1*) were verified in four cell lines with statistically significant higher average fold increases observed for three of these genes in the less sensitive cells (MCF7 and T47D) relative to the sensitive cells (SKBR3 and MDA-MB-231) after 48 hours exposure to atorvastatin (Figure 1B). Very similar results were seen following 24 hours statin treatment (Supplementary Figure S2). In agreement with the differential upregulation of *HMGCR* mRNA, immunoblot experiments revealed that, atorvastatin treatment also substantially increased HMGCR protein levels, with an average fold increase of 9.3 vs 2.6 in the less-sensitive cells (MCF7, T47D and BT474) relative to the sensitive cells (SKBR3 and MDA-MB-231) (Figure 1C-1E), directly mirroring the mRNA changes.

### Basal expression of cholesterol biosynthesis genes is associated with atorvastatin sensitivity in breast cancer cell lines and primary tumors

**Figure 2:**
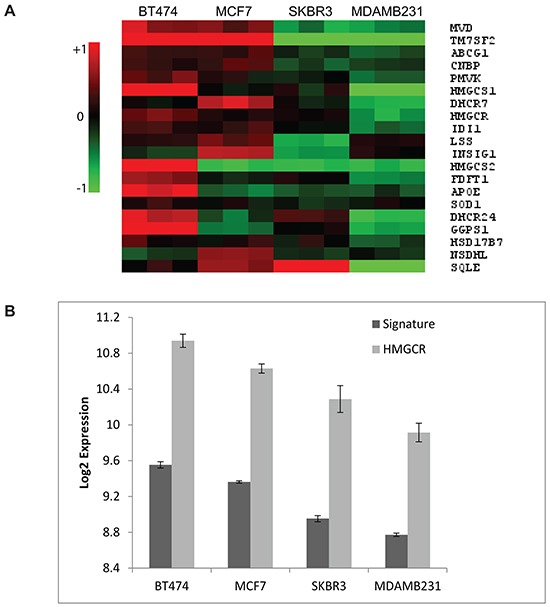
Cholesterol biosynthesis genes are differentially expressed in untreated breast cancer cells **A.** Basal expression of the “cholesterol biosynthesis signature” in breast cancer cell lines. Red in the heat map represents upregulation and green represents downregulation. **B.** A direct correlation between the basal expression of the signature with the HMGCR transcript is observed. Data plotted in (B) are the mean Log2 expression +/− the standard deviation from the three biological replicates per cell line. Two tailed t-tests comparing the expression of the signature and HMGCR between the statin sensitive (MDA-MB-231 and SKBR3) and the in-sensitive (BT474 and MCF7) cells resulted in P=0.003 and P=0.015 for the signature and HMGCR respectively.

To further explore the significance of the differential upregulation of cholesterol biosynthesis genes following atorvastatin treatment as observed in the cell line panel, we investigated if statistically significant differences in the expression of genes in this pathway pre-existed at baseline; putting the hypothesis that this pathway is dysregulated in some cells and may be attributed to the differential response to atorvastatin treatment to the test. A supervised SAM analyses was performed, comparing the baseline expression levels of genes included in the cholesterol biosynthesis process (GO:006695) between the atorvastatin sensitive (MDA-MB-231 and SKBR3) and the less-sensitive (MCF7 and BT474) cells. Twenty genes (henceforth referred to as the “cholesterol biosynthesis signature”), including *HMGCR*, displayed a significantly lower expression in the atorvastatin sensitive cells relative to the less-sensitive cells (FDR<0.05; Figure 2A). Interestingly, the expression of the *HMCGR* transcript (Figure 2B) and protein (Figure 1C) were found to consistently follow the expression of the signature, suggesting that this single gene may serve as a surrogate for the signature.

**Figure 3:**
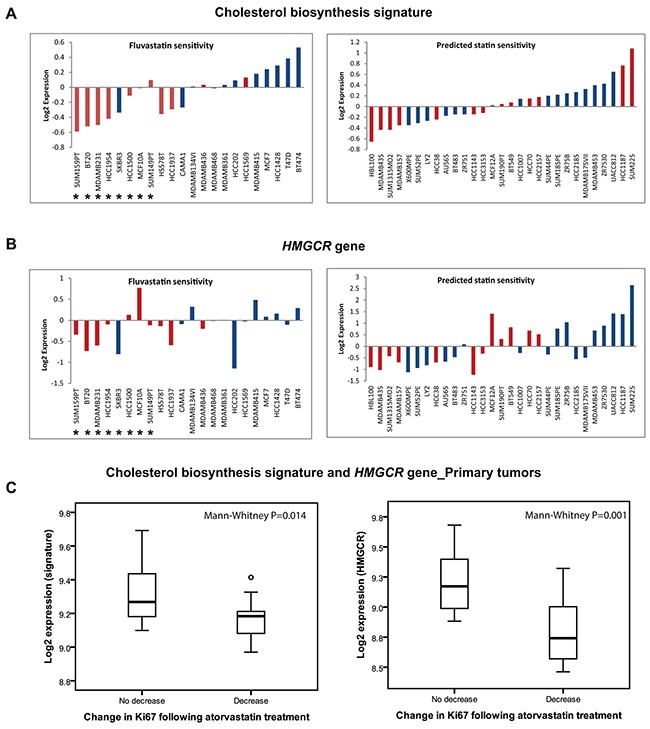
The “cholesterol biosynthesis signature” can predict response to statin treatment in cell lines and primary tumors **A.** Low expression of the “cholesterol biosynthesis signature” or *HMGCR* **B.** is associated with fluvastatin sensitivity in breast cancer cell lines. Fluvastatin sensitive cells (marked *) as characterized by the study of Goard et al [6]. The cell lines are coloured according to their molecular subtypes (red; basal-like and blue; luminal). **C.** Low basal expression of the “cholesterol biosynthesis signature” or *HMGCR* is also associated with reduction in tumor proliferation following statin treatment in clinical breast cancer samples.

To independently verify if the expression of the “cholesterol biosynthesis signature” or *HMCGR* was associated with the sensitivity to statin treatment in a larger panel of breast cancer cell lines, we utilised an external gene expression dataset including 51 breast cancer cell lines [13, 14], 22 of which had been previously characterised for fluvastatin sensitivity [6]. In that study [6], fluvastatin sensitivity (after 72 hours of drug exposure) was set at MTT_50_≤ 20 μM. The median expression of the “cholesterol biosynthesis signature” was compared between cell lines. Interestingly, 7/8 fluvastatin sensitive and 3/14 fluvastatin resistant cell lines displayed low expression of the cholesterol biosynthesis signature (Figure 3A). Very similar results were seen when the *HMGCR* transcript was utilised (Figure 3B). Although sensitivity to fluvastatin had been directly tested in proliferation assays for only 22 of the 51 cell lines, low expression of the cholesterol signature; suggesting statin sensitivity, was also observed in other un-tested basal-like or luminal cells. In general, a higher proportion of basal-like cell lines (54%) displayed low expression of the signature but a considerable number of luminal cell lines (32%) also displayed low expression indicating that both luminal and basal-like breast cancers are potentially sensitive to statin treatment.

Our group has previously reported results from a “window-of-opportunity” trial showing that two weeks of treatment with a high dose of atorvastatin (80 mg/day) was capable of decreasing proliferation (i.e. Ki67 expression) in a subset of tumors [10]. Paired tumor biopsies collected before the commencement of atorvastatin treatment and at time of surgery after completing the 2 weeks course of statin treatment have also been subjected to whole genome transcriptional profiling [12]. We compared the expression of our “cholesterol biosynthesis signature” at baseline between the tumors that responded with a decrease in proliferation (i.e. atorvastatin sensitive; n=15) compared to tumors that showed no change or increased proliferation (i.e. atorvastatin insensitive; n=10). The baseline expression of the signature was found to be significantly lower in the group of tumors with a reduced proliferation index following statin treatment (Mann-Whitney P=0.014; Figure 3C). A similar trend was once again noted for *HMGCR* only (Mann-Whitney P=0.001; Figure 3C).

Evaluating HMGCR by transcriptional profiling and by immunohistochemistry has yielded equivocal results. Given that high basal HMGCR protein expression was also reported to be a potential statin-treatment predictive marker in this window-of-opportunity trial [10], the finding that low mRNA expression is associated with atorvastatin response in the same material is therefore unexpected. To address this discordant result, we investigated if HMGCR mRNA tracts protein expression in the tumors from this window-of-opportunity trial and found that HMGCR mRNA expression was inversely-correlated with protein expression as quantified by the anti-HMGCR polyclonal antibody (HPA008338) that was used for IHC staining as reported in the window trial [10] (Supplementary Figure S3: r= −0.36; P=0.077 and r= −0.18; P=0.379 for pre- and post-atorvastatin samples respectively). The fact that mRNA and protein expression as measured by this polyclonal antibody are inversely correlated may partially explain the disparate findings between the previous [10] and the current study, since the patients presenting with low mRNA expression are largely represented among those considered to express high HMGCR by the polyclonal antibody.

### The expression of the “cholesterol biosynthesis signature” is associated with the prognosis following primary breast cancer diagnosis

**Figure 4:**
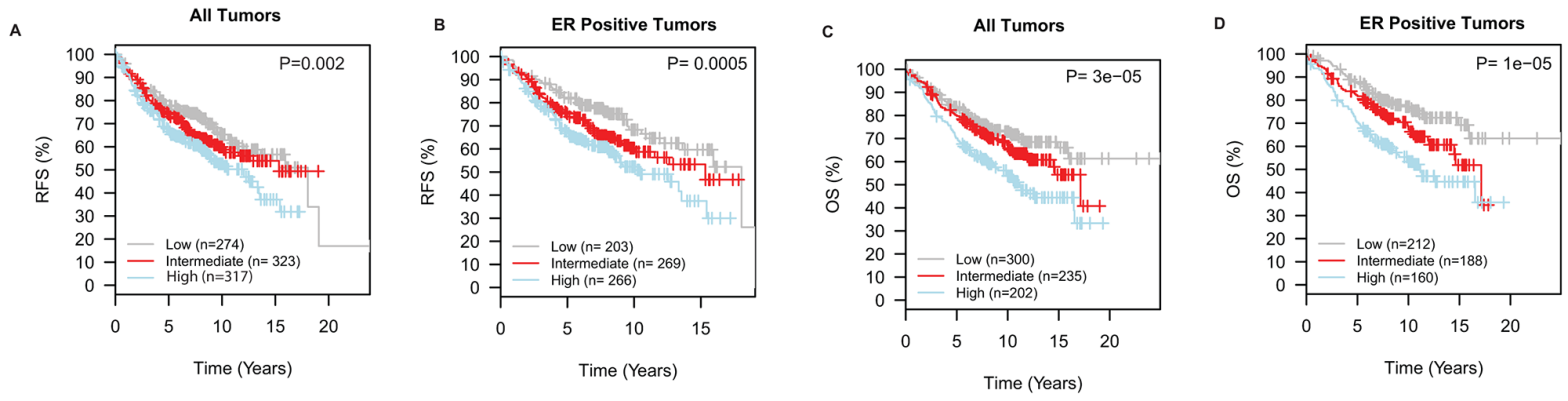
Dysregulation of cholesterol biosynthesis is associated with the prognosis following primary breast cancer diagnosis Low expression of the “cholesterol biosynthesis signature” is associated with a longer recurrence-free survival, RFS **A.** and **B.** and overall survival, OS **C.** and **D.**

Dysregulation of cholesterol metabolism via the mevalonate pathway has been highlighted to play a role in malignant transformation and tumor progression [15, 16]. Recently, activation of endogenous cholesterol biosynthesis was implicated in the development of resistance to aromatase inhibitors in breast cancer [17]. Also, the prognostic relevance of genes/proteins involved in this pathway has been previously investigated in breast cancer [15, 17–20], although the data are controversial. In the independent validation cohort of primary breast cancers, we explored if dysregulation of cholesterol biosynthesis, as captured by our signature, was significantly associated with primary tumor pathological features and patients' survival. For survival analyses, the cohort was split into three groups using the tertiles of the expression of “the cholesterol biosynthesis” signature as cut points. Low expression of the signature was significantly associated with ER negativity and the basal-like molecular subtype in cell lines (Figures 3A-3B) and primary tumors (Supplementary Figure S4A-S4B). Notably, within the luminal subtypes, luminal A tumors displayed lower expression of “the cholesterol biosynthesis signature” relative to the luminal B tumors (Supplementary Figure S4B). Importantly, high expression of “the cholesterol biosynthesis signature” was associated with a significantly shorter recurrence-free survival (RFS, Log-rank P<0.01) and overall survival (OS, Log-rank P<0.001), especially in ER positive tumors (Figure 4). A similar trend was once again observed when *HMGCR* was considered alone (Supplementary Figures S4C-S4F). Moreover, the “cholesterol biosynthesis signature” remained an independent prognostic factor for RFS and OS in multivariable analyses adjusting for conventional prognostic factors in primary breast cancer such as age at primary diagnosis, ER status, tumor histological grade, nodal involvement and size of the primary tumor (Table 1). Independent analyses in the TCGA cohort (Supplementary Figure S5) confirmed that high expression of the cholesterol biosynthesis signature was associated with an inferior overall survival (Log-rank P=0.04).

**Table 1:** Multivariable Cox proportional hazards analyses for recurrence-free survival (RFS) and overall survival (OS). Separate analyses are shown for all patients and for the subset of patients with ER positive tumors. P<0.05 was considered significant

Factor	Recurrence-free survival (RFS)						Overall survival (OS)					
	All tumors			ER positive tumors			All tumors			ER positive tumors		
	HR	CI	*P*	HR	CI	*P*	HR	CI	*P*	HR	CI	*P*
**Cholesterol Signature** (High vs Intermediate)	0.80	0.62-1.04	0.09	0.86	0.64-1.1	0.29	0.84	0.61-1.1	0.26	0.93	0.66-1.3	0.71
(High vs Low)	0.64	0.48-0.85	0.002	0.62	0.44-0.89	0.008	0.62	0.45-0.88	0.003	0.62	0.42-0.92	0.02
**ER status** (Pos vs Neg)	0.76	0.56-1.03	0.07	n.a	n.a	n.a	0.80	0.58-1.1	0.18	n.a	n.a	n.a
**Nodal status** (Pos vs Neg)	0.69	0.53-0.90	0.006	0.72	0.54-0.96	0.03	0.46	0.35-0.60	<0.0001	0.47	0.34-0.65	<0.0001
**Histological grade** (3 vs 1 and 2)	0.98	0.75-1.3	0.88	1.1	0.83-1.5	0.43	1.3	0.94-1.7	0.11	1.4	1.02-2.0	0.04
**Age at diagnosis** (>50 vs ≤50)	0.77	0.60-0.99	0.04	0.74	0.56-0.98	0.03	1.5	1.1-1.9	0.008	1.6	1.1-2.2	0.009
**Tumor size** (>2 cm vs ≤2 cm)	1.71	1.4-2.2	<0.0001	2.1	1.6-2.7	<0.0001	1.8	1.4-2.3	<0.0001	1.8	1.3-2.5	**0.002**

HR; hazard ratio, CI; confidence interval, *P*; P-value, Pos; positive, Neg; negative, n.a; not applicable

## DISCUSSION

The appreciable molecular and clinical heterogeneity of breast cancer warrants that for every novel drug showing efficacy against this disease, potential treatment predictive biomarkers should be identified to enable the precise selection and specific treatment of only patients who may derive benefit from the therapy. In this study, we aimed to uncover transcriptional features associated with statin response in breast cancer. We found that, in addition to ER expression, one of the strongest discriminant between statin sensitive and less-sensitive breast cancer cells at the transcriptional level was the expression of genes involved in the cholesterol biosynthesis pathway. Following statin treatment, the less-sensitive cells were capable of strongly inducing the expression of genes in this pathway via the normal negative feedback loop resulting from the statin-induced inhibition of HMGCR, the rate limiting enzyme and key regulator of the mevalonate pathway [21]. This classical response was however weaker in the sensitive cells, suggesting that these cells may possess an inherent defect in this pathway. The inability to significantly upregulate mevalonate pathway genes in response to the inhibition of HMGCR has also been reported in statin-sensitive multiple myeloma cells [11], indicating that this feature may be common across many tumor types and further suggests that our “cholesterol biosynthesis signature” may serve as a useful biomarker for statin sensitivity in not only breast cancer but other cancer types.

The general conclusion from previously conducted *in-vitro* studies is that ER negative breast cancer cells are more sensitive to statin treatment [5, 6, 8, 22] and by inference; these studies suggest that breast cancer patients with ER negative tumors are most likely to derive benefit from statin treatment. Consistent with our findings, Goard CA and colleagues [6] also reported that sensitivity of breast cancer cell lines to fluvastatin treatment was inversely correlated with basal HMGCR mRNA and protein expression levels. This study did not reveal a statistical significant association between sensitivity to treatment and the statin-induced feedback induction of HMGCR at the mRNA level, although induction of HMGCR protein was found to be correlated with fluvastatin sensitivity. Despite this discrepancy, the results from this study largely align with our data, lending more support to the proposition that the basal levels of our “cholesterol biosynthesis signature” (or HMGCR) and not the statin-induced feedback regulation of HMGCR per se, is a better predictor of statin sensitivity. As mentioned previously, there is strong epidemiological evidence supporting a positive association between statin use and an extended recurrence-free survival, which is also true for patients with ER positive tumors [1, 4]. Moreover, the oxysterol; 27-hydroxycholesterol, a cholesterol metabolite and an endogenous selective estrogen receptor modulator capable of promoting the autonomous growth of ER positive breast cancer [7, 23, 24], is thought to be the biochemical link between the elevated risk of ER positive breast cancers in women with obesity and/or hypercholesterolemia [7]. In this regard, the indirect effect of statin treatment which results in decreasing the systemic levels of total cholesterol, LDL-cholesterol and by inference 27-hydroxycholesterol may thus inhibit tumor proliferation and prevent or delay the onset of metastatic disease [7], providing a molecular basis for the benefits of using statins to control ER positive breast cancers. Since it is unlikely that the extrahepatic concentrations of most orally administered statins will reach the doses utilised *in vitro* and also considering the possibility of other confounding effects due to the reduction of cholesterol precursors required for protein prenylation and farnesylation especially after prolonged treatment (48h) of cells *in vitro* with statins, the effects on the tumor would likely be due to reduced circulating cholesterol (or 27-hydroxycholesterol) levels. Regardless, the gene signature generated *in vitro* is predictive of decreased proliferation in tumors from statin treated patients. We observed that more than half of basal-like and a third of luminal cell lines displayed low expression of the signature. Of the 25 primary tumors included in the analyses evaluating the predictive value of our “cholesterol biosynthesis signature” (and *HMGCR*), only two were ER negative and the proliferation index in these two tumors decreased following statin treatment. Importantly, the expression of the “cholesterol biosynthesis signature” and *HMGCR* were significantly lower in the group of 15 tumors responding with a decrease in proliferation, 13 of which were ER positive. Of note, in the clinical trial conducted by Garwood and colleagues [9], evaluating the effect of fluvastatin on breast cancer proliferation, the proliferation index reduced in 20/29 tumors, the majority of which were ductal carcinomas *in situ*. However, in that study [9], no statistically significant difference was noted between tumors stratified by ER status, although the median reduction in proliferation trended to be higher in the ER negative group. Taken together, our results while confirming the strong association between ER negativity (basal-like molecular subtype) and statin sensitivity also reveals that dysregulation of cholesterol biosynthesis is a marker of statin response, independent of ER status. Further validation of the treatment predictive potential of this signature is necessary in a larger and more representative cohort of breast cancer patients.

The prognostic relevance of genes involved in cholesterol biosynthesis has been explored in breast cancer with equivocal results. In agreement with our results, high expression of the mRNA of *HMGCR* and other sterol-response genes, analysed separately, correlated with poor prognosis in breast cancer patients [15]. Similarly, a recently published multigene signature also enriched for cholesterol biosynthesis genes [17], which was implicated in resistance to aromatase inhibition, was shown to have prognostic relevance in primary breast cancer. Low expression of this signature was associated with a favourable outcome, consistent with our results. The prognostic importance of HMCGR evaluated by immunohistochemistry (IHC) has yielded opposite results in relation to survival. High HMGCR expression was associated with either favourable tumor pathological features and/or a better breast cancer survival [18–20]. The reason for this controversy remains unclear but specificity of antibodies against HMGCR has been suggested to be a probable explanation [15]. This discordance between HMGCR mRNA and protein quantification was again highlighted by this study. We observed that low basal *HMGCR* mRNA expression is predictive of reduction in tumor proliferation after atorvastatin treatment contrary to the previous data showing that high protein expression was associated with the likelihood of decreasing tumor proliferation by statin response. The inverse correlation between mRNA and protein expression as evaluated in the previous study [10] may provide an explanation for the significant associations with the change in tumor proliferation reported in both the previous [10] and the present study. In this study, we evaluated HMGCR protein expression using a new monoclonal antibody which specifically recognised the human HMGCR at the predicted 97 kDa molecular weight and which reliably detected the upregulation of HMGCR following atorvastatin treatment in different cell line models (Figure 1C and 1D). Restricted by the absence of tumor material, we were only able to perform IHC using this monoclonal antibody on the post treatment samples from the window-of-opportunity trial [10]. Similar to the cell lines, a positive correlation was noted between HMGCR protein and mRNA expression *(r=0.3, p=0.18),* warranting further studies to re-evaluate the statin treatment predictive and prognostic relevance of HMGCR in breast cancer using this monoclonal antibody. Notwithstanding, the consistency of the mRNA data from three independent studies including the current investigation lends more support to our present recommendation that low expression of cholesterol biosynthesis genes and *HMGCR* is associated with favourable prognostic features and survival especially in ER positive breast cancer and may predict sensitivity to statin treatment. Sub-analyses of the prognostic importance of the signature and *HMGCR* in ER negative tumors did not reach statistical significance (data not shown) probably due to the less heterogeneous expression of these genes in ER negative tumors. A trend towards a more inferior outcome was however noted for tumors within the top tertile (highest expression) within this sub-group.

Statin therapy is an attractive addition for the clinical management of breast cancer but for successful clinical implementation, careful selection of patients to receive treatment is warranted. While confirming that dysregulation of cholesterol biosynthesis is associated with breast cancer prognosis, our data further suggest a novel transcription-based biomarker with a promising potential for identifying the patients most likely to derive benefit from the addition of cholesterol lowering medications to their therapeutic regimen for controlling breast cancer.

## MATERIALS AND METHODS

### Cell lines, cell culture and treatments

MCF7, BT474, SKBR3 and MDA-MB-231 cell lines were purchased from the American Type Culture Collection (Rockville, MD) and were maintained at 37 °C in a humidified chamber with 5% CO_2_. MCF7, BT474, SKBR3 were cultured in Dulbecco's Modified Eagle's Medium (DMEM):Ham's F-12 1:1 and MDA-MB-231 was grown in RPMI-1640. All media were supplemented with 10% fetal bovine serum (FBS), 2 mmol/L l-glutamine, 20 units/ml penicillin, and 20 μg/ml streptomycin. Atorvastatin calcium salt (Sigma-Aldrich) was dissolved in DMSO (Sigma) for *in vitro* experiments. Cells were treated with atorvastatin (1-100 μM) for 72 hours after which the effect of the treatment on cell proliferation was measured using the xCELLigence Real-Time Cell Analyzer (ACEA Bioscience, Inc).

### RNA extraction and gene expression microarrays

After treating MCF7, BT474 and SKBR3 cells with 5 μM atorvastatin and MDA-MB-231 cells with 1 μM of atorvastatin for 48 hours, total RNA was extracted using the QIAGEN RNeasy kit (QIAGEN, Valencia, CA) according to the manufacturer's instructions. The integrity of the RNA was assessed on an Agilent 2100 Bioanalyzer (Agilent, Santa Clara, CA), and the quantity of RNA in each sample was determined using a NanoDrop ND-1000 (NanoDrop Products, Wilmington, DE). RNA was amplified, labeled and hybridized unto the Human HT-12 v4.0 Expression BeadChips according to the Illumina-recommended protocol (Illumina, Inc.). All samples were processed in one batch.

### Clinical sample material for gene expression validation

Microarray data from 25 patients with invasive primary breast cancers treated with high dose atorvastatin for two weeks in a pre-surgical window-of-opportunity trial (GSE63427) [10, 12] and a second cohort including a collection of 51 breast cancer cell lines and 1,881 primary breast tumors [14] were used for validation studies. The prognostic relevance of the cholesterol biosynthesis signature was independently verified in a subset of 661 invasive breast carcinomas from the TCGA project (update September 2013). RNAseq v2 level 3 data for the 661 tumors included in this analysis were processed as previously described [25].

### Gene expression data analysis

Microarray data were pre-processed and quantile-normalized using the GenomeStudio Software V2011.1 and BioArray Software Environment (BASE) V3.4.1 respectively. Probe sets with signal intensities below the median of the negative control intensity signals in ≥80% of the samples were excluded. The Illumina probes were re-annotated using the R package illuminaHumanv4.db. Replicate probe sets were merged by the median of signal intensity values. The normalized gene expression data is publicly available at the NCBI's Gene Expression Omnibus (GEO) database as part of the GSE63427 series.

Significance Analysis of Microarrays (SAM) analyses [26] were performed to identify genes differentially altered by atorvastatin treatment in each cell line and a false discovery rate (FDR) <5% was used as a cut-off for significance. The differentially altered genes were functionally annotated and significantly deregulated biological processes and pathways were uncovered using the DAVID Functional Annotation Tool [27, 28] and the ToppGene Suite [29]. The Homo sapiens background was used as default and correction for multiple hypothesis testing was done using the FDR. Finally, a two class SAM analysis was performed to compare the basal expression of genes involved in the cholesterol biosynthesis process (GO:006695) between statin sensitive and less-sensitive cells, resulting in the identification of a 20 gene “cholesterol biosynthesis signature” associated with response to statin treatment. The treatment predictive value of this signature was independently investigated in the two validation cohorts. The Mann-Whitney test was applied to assess the difference in the median expression of the “cholesterol biosynthesis signature” between statin sensitive and resistant tumors.

#### RT-qPCR gene expression analysis

Following treatment for 24 hours and 48 hours with an equivalent dose of atorvastatin per cell line as used for microarray experiments, the statin-induced feedback response for four selected genes included in our cholesterol signature (*HMGCR*, *HMGCS1*, *MVD*, and *INSIG1*) was validated in four breast cancer cell lines using RT-qPCR. Briefly, total RNA was reverse-transcribed into cDNA (High-Capacity cDNA Reverse Transcription Kit, Thermo Fisher Scientific) which was used as a template in the qPCR reaction. Pre-designed primers and hydrolysis probe assays were used to amplify the genes of interest (Applied Biosystems: *HMGCR*; HS00168352_M1, *HMGCS1*; HS00266810_m1, *MVD*; HS00964563_g1, *INSIG1*; HS01650979_m1, and *ACTB*; HS99999903_m1). Expression measurements were then made by comparing cycle-threshold (CT) of the amplicons of interest to an internal reference amplicon in the house-keeping gene *ACTB*. All samples were run in triplicate and no template controls were included in each run. Data was analysed using the 2 (-DeltaDeltaC(T)) method [30]. The final gene expression measurements represent the mean and s.d. of three biological replicates, each composed of at least two independent experiments.

### Survival and multivariable Cox-regression analyses

Kaplan-Meier plots were generated and the Log-rank test was used to check for statistically significant survival differences between patient groups stratified according to the expression of the genes associated with statin response. Cox-proportional hazards models were used to evaluate the independent prognostic significance of the genes associated with statin response, adjusting for conventional prognostic factors. P-values correspond to two-sided statistical tests and values <0.05 were considered significant. The REMARK guidelines for reporting prognostic biomarker studies were followed [31].

### Western blotting

Cells were washed with ice-cold PBS and lysed with ice-cold RIPA buffer (150 mM NaCl, 1% NP-40, 0.5% 0.5% Na-deoxycholate, 0.1%sodium dodecyl sulfate, 10 mM TrisHCl pH 8) supplemented with phosphatase and protease inhibitor cocktails (Complete Mini and PhosphoStop, F. Hoffmann-La Roche Ltd). Lysates were centrifuged at 16,000g for 30 minutes at 4°C, supernatants were collected and the protein concentration was determined using the Pierce BCA Protein Assay Kit (Thermo Scientific Inc.). Equal amounts (20 μg) of protein were resolved on 4-12% Tris-HCL gels (Bio-Rad), and electrophoretically transferred to Immobilon-PVDF transfer membranes. Membranes were blocked for 1 hour in 5% non-fat milk in Tris-Buffered Saline (TBS)-Tween and then hybridized using a primary monoclonal antibody against HMGCR (AMAb90619, Atlas Antibodies, Sweden) at a dilution of 1:1000 in 5% non-fat milk TBS-Tween. β-actin was used as a loading control (1:1000, Cell Signalling Technology). Horseradish peroxidase (HRP)-conjugated secondary antibodies (1:10,000) were also prepared in 5% non-fat milk in TBS-Tween. Protein–antibody complexes were detected by chemiluminescence with the Clarity^™^ ECL Western Blotting Substrate (Bio-Rad) and images were captured and the relative HMGCR protein expression was quantified with the CHEMIDOC^™^ MP imaging system (Bio-Rad).

## SUPPLEMENTARY FIGURES


