# Supplementary material for: High expression of cholesterol biosynthesis genes is associated with resistance to statin treatment and inferior survival in breast cancer
Source: Oncotarget. 2016 Jul 21;7(37):59640–51. doi: 10.18632/oncotarget.10746 (PMC5312337; doi:10.18632/oncotarget.10746)
Supplement: Supplementary file 1 [file oncotarget-07-59640-s001.pdf]

High expression of cholesterol biosynthesis genes is associated with resistance to statin treatment and inferior survival in breast cancer

SUPPLEMENTARY FIGURES

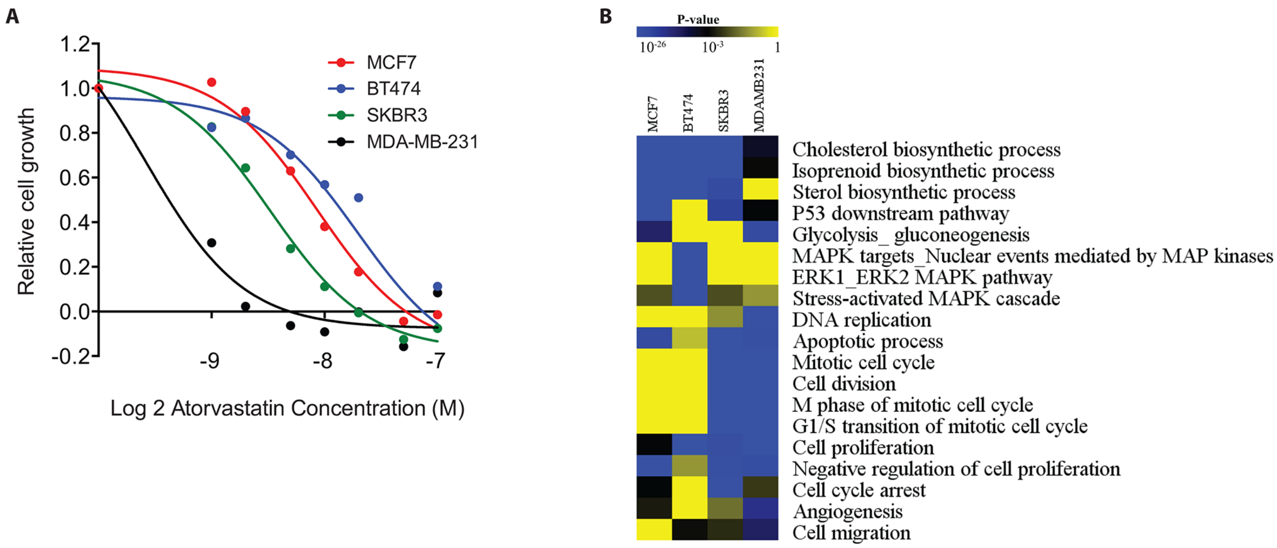

Supplementary Figure S1: Effect of statin treatment of breast cancer cells. Atorvastatin treatment differentially effects cell proliferation **A.** and dysregulation of specific biological processes and pathways **B.** in breast cancer cell lines. Data plotted in Supplementary Figure 1A are the mean of three independent experiments. Data presented in supplementary Figure 1B in part overlap with a Figure 4B in our previous publication [12] which partially reported on the discovery microarray data that was used in this study.

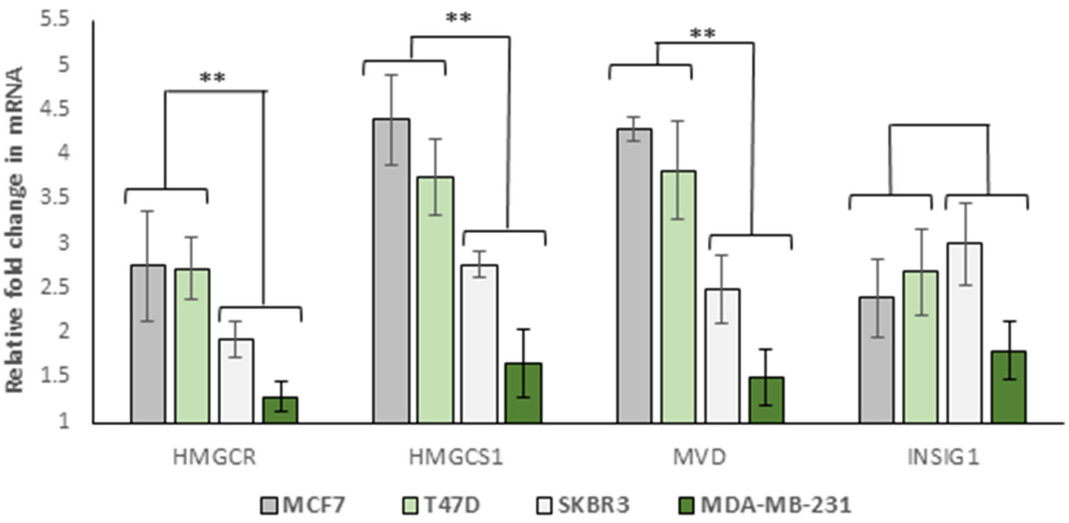

Supplementary Figure S2: RT-qPCR validation of the statin-induced feedback upregulation of four genes in the cholesterol biosynthesis pathway. Fold change of mRNA expression after 24 hours of atorvastatin treatment are shown. The average fold change in each gene was compared between the less sensitive (MCF7 and T47D) and the sensitive (SKBR3 and MDA-MB-231) cell lines. * represents P<0.05 and ** represents P<0.01 respectively.

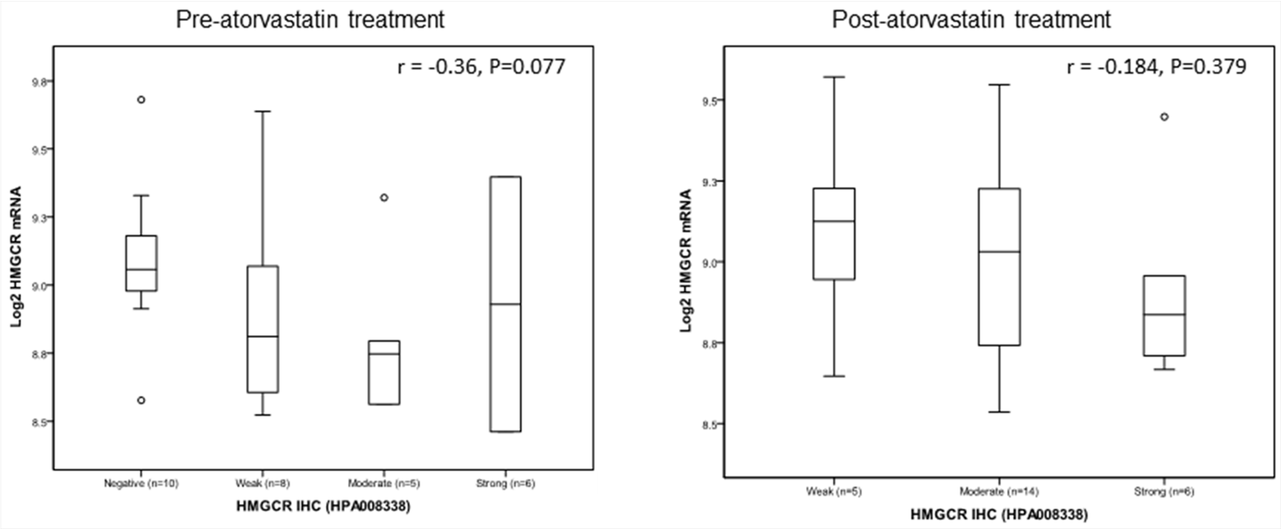

Supplementary Figure S3: Correlation between HMGCR mRNA and protein expression in primary breast tumors. A negative correlation was noted between mRNA expression and protein expression quantified by the anti-HMGCR polyclonal antibody HPA008338.

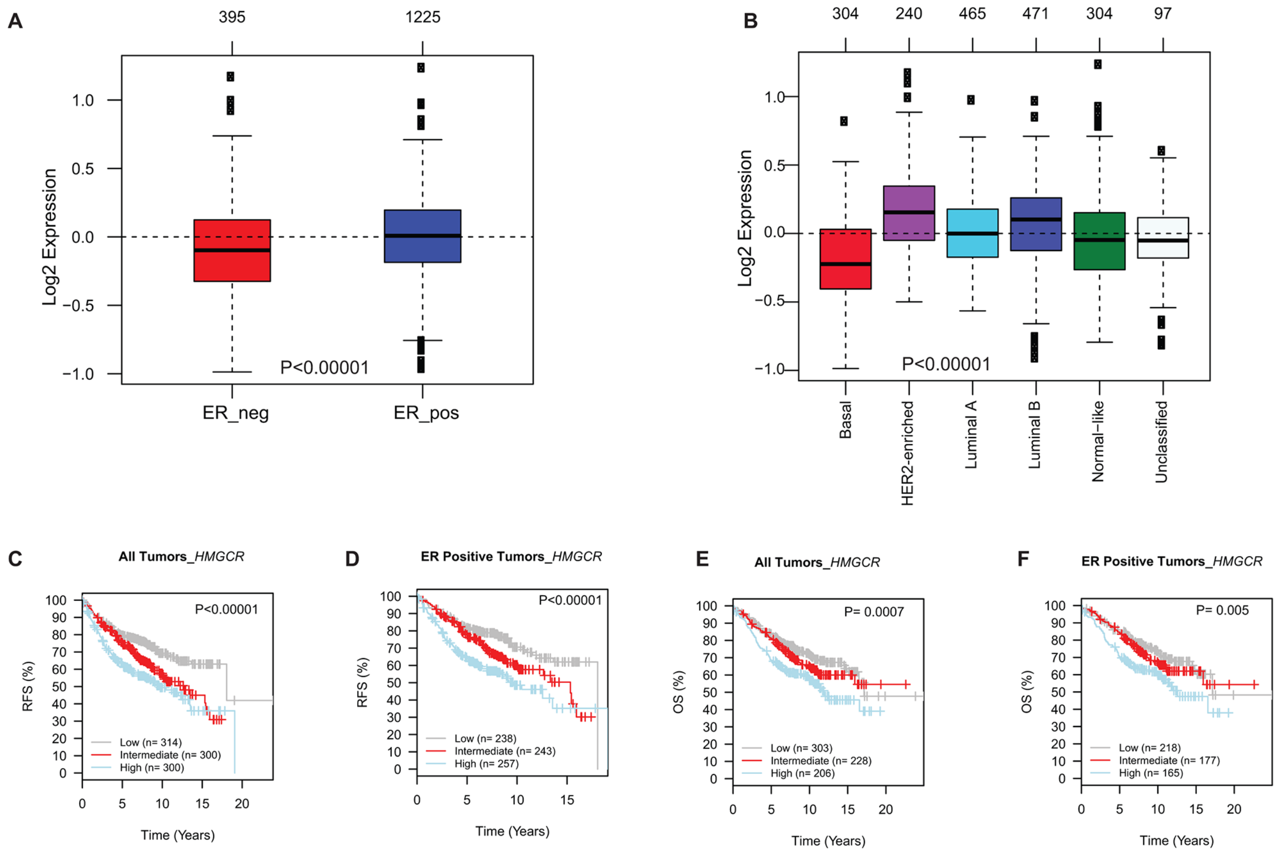

Supplementary Figure S4: The basal expression of the "cholesterol biosynthesis signature" is significantly heterogeneous in primary tumors (A and B). Low expression of *HMGCR* is associated with longer recurrence-free survival, RFS **C.** and **D.** and overall survival, OS **E.** and **F.**

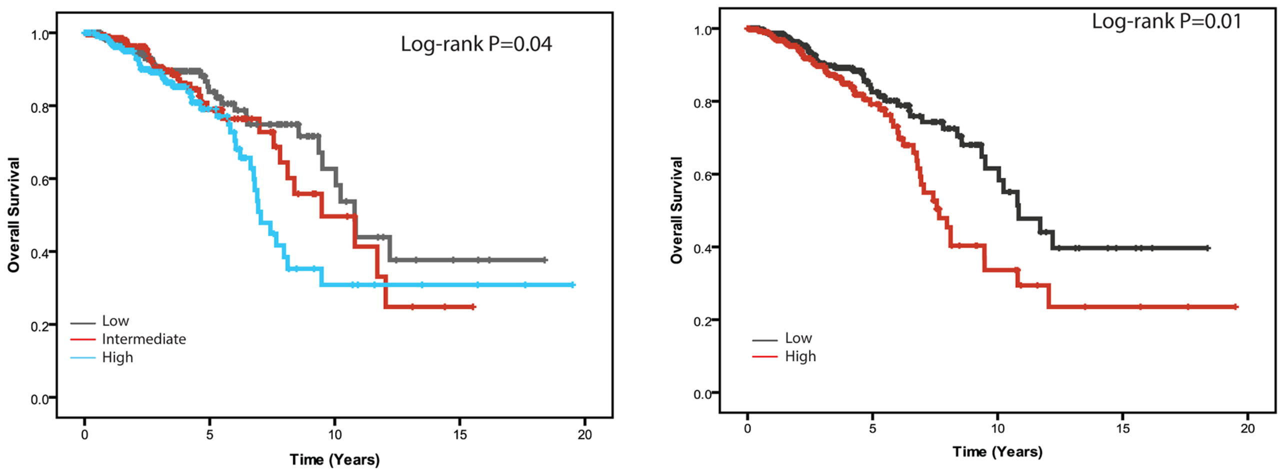

Supplementary Figure S5: High expression of the "cholesterol biosynthesis signature" is significantly associated with an inferior overall survival in the TCGA cohort. Patients were stratified by the **A.** tertiles and the **B.** median expression of the signature.
